# Is It Time to Consider Hydroxyurea as a Potential Therapeutic Option in the Management of Malignant Pertussis – A Case Report and Literature Review

**DOI:** 10.7759/cureus.87648

**Published:** 2025-07-10

**Authors:** Abdullah I Alsharif, Yousef DakheelAllah Alatawi, Abeer Mohammed M Alanazi

**Affiliations:** 1 Pediatrics Infectious Diseases, Maternity and Children Hospital, Tabuk, SAU; 2 Pediatric Hematology Oncology, Maternity and Children Hospital, Tabuk, SAU; 3 Pediatrics, Maternity and Children Hospital, Tabuk, SAU

**Keywords:** bordetella pertussis, hydroxyurea, hyperleukocytosis (hl), leukoreduction therapy, malignant pertussis

## Abstract

Malignant pertussis is a life-threatening condition in infants, for which no effective evidence-based treatment strategies have been established, owing to the rarity of the condition. We present the case of a four-month-old infant with paroxysmal coughing spells followed by a whooping sound and shortness of breath with a leukocyte count of 41.82 × 10^9^/L and lymphocytosis (32.25 × 10^9^/L). A diagnosis of malignant pertussis, based on the severe paroxysmal cough, significant lymphocytosis, and progressive respiratory symptoms, was established, and treatment was initiated with azithromycin and hydroxyurea (20 mg/kg/day) under intensive supportive care. The leukocyte count gradually decreased over five days, and the infant recovered completely after seven days of hospitalization. Notably, the infant did not develop pulmonary hypertension and did not require leukapheresis or any transfusion support during the course of treatment. The case report provides valuable information regarding the presentation of such cases, their diagnosis, and the effectiveness of hydroxyurea as an alternative to whole blood transfusion and leukapheresis. Hydroxyurea is a promising treatment in infants with malignant pertussis and effectively induces a gradual but progressive decline in leukocytosis. Early administration in hemodynamically stable patients may reduce the need for invasive procedures; however, its use in more severe cases requiring urgent leukoreduction may be limited due to the delayed onset of action. This case adds to the limited number of reported instances where hydroxyurea was successfully used as a noninvasive leukoreductive option in malignant pertussis.

## Introduction

Pertussis (commonly known as whooping cough) is a highly contagious respiratory illness caused by the bacterium *Bordetella pertussis* [1]. Despite widespread vaccination programs, pertussis remains a significant cause of morbidity and mortality among infants, particularly those under one year of age. In 2014, approximately five million pertussis cases and 85,900 pertussis-related deaths were reported globally in this age group [2].

A resurgence of pertussis has been observed in several countries, which is attributed to multiple factors, including the waning immunity conferred by the acellular pertussis vaccine, antigenic variation of *B. pertussis*, and the emergence of vaccine escape mutants [1,3]. These trends underscore the ongoing public health challenge posed by pertussis, even in vaccinated populations.

A particularly severe form of the disease, malignant pertussis, may develop in a subset of infants. It is characterized by a rapidly progressive clinical course with features such as hyperleukocytosis (often >50-100 × 10^9^/L), pulmonary hypertension, pneumonia, cardiogenic shock, and respiratory failure [4,5]. The diagnostic features typically include a combination of: (1) severe paroxysmal cough or apnea; (2) marked leukocytosis, especially lymphocytosis; and (3) signs of cardiopulmonary compromise such as pulmonary hypertension or shock [6,7]. The condition carries a high case fatality rate, often despite intensive supportive care.

Treatment options for malignant pertussis have included antimicrobial therapy (e.g., macrolides), leukoreductive therapies such as leukapheresis or exchange transfusion, and aggressive management of pulmonary hypertension using agents such as inhaled nitric oxide or sildenafil [6,7]. However, there is currently no universally accepted evidence-based treatment algorithm, due to the rarity of the condition and the logistical limitations of conducting randomized controlled trials.

In this context, case reports play a critical role in expanding clinical knowledge and guiding practice. The current report presents a case of malignant pertussis in an infant successfully managed with azithromycin and hydroxyurea, a cytoreductive agent that may represent a less invasive alternative to traditional leukoreductive techniques. This case adds to the limited but growing body of literature suggesting a potential therapeutic role for hydroxyurea in gradually controlling leukocytosis in hemodynamically stable infants with malignant pertussis.

## Case presentation

A four-month and 13-day-old previously healthy full-term female infant, born via normal vaginal delivery with no prior hospital admissions, was brought to the emergency department with a 10-day history of paroxysmal coughing spells followed by a whooping sound and episodes of central cyanosis. The mother reported associated shortness of breath, but no fever, vomiting, or feeding difficulties. The infant was exclusively breastfed and had received only the birth dose of hepatitis B vaccine (according to the Saudi national schedule). There was a positive history of contact with household members suffering from upper respiratory tract infections.

**Figure 1:**
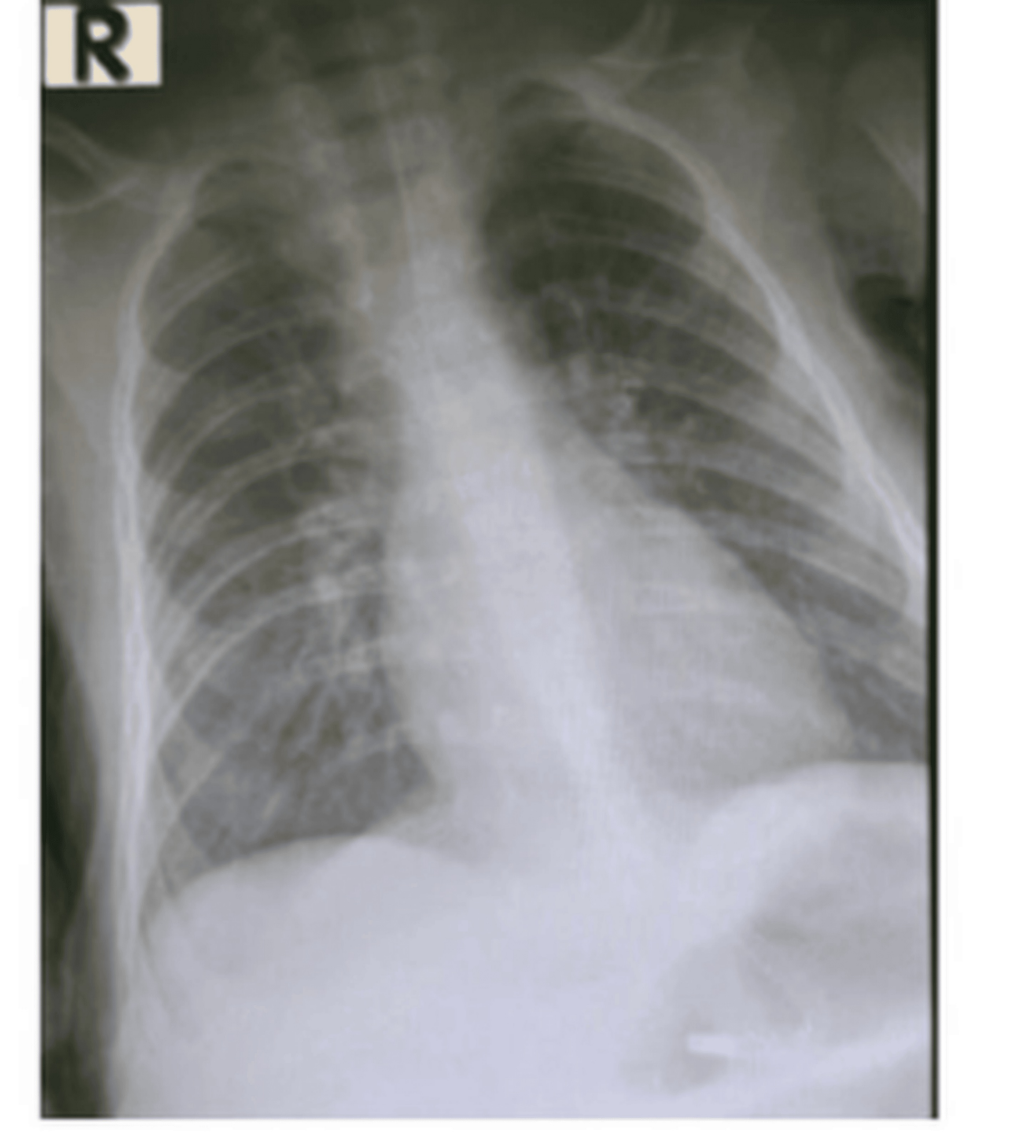
Chest radiograph of the infant with malignant pertussis The image demonstrates diffuse bilateral perihilar infiltrates and hyperinflation.

On arrival to the ED, the infant experienced three witnessed episodes of paroxysmal cough with central cyanosis. Vital signs were heart rate at 164 bpm, respiratory rate at 47 breaths/min, BP of 88/45 mmHg, and normal temperature. Chest auscultation revealed diffuse bilateral crepitations. Chest X-ray demonstrated diffuse bilateral perihilar infiltrates and hyperinflated lungs without focal consolidation (Figure 1).

Initial laboratory investigations showed a white blood cell (WBC) count of 41.82 × 10^9^/L (reference range: 5-15 × 10^9^/L) with marked lymphocytosis (32.25 × 10^9^/L; reference range: 5-15 × 10^9^/L) and a negative C-reactive protein (CRP). Nasopharyngeal swab polymerase chain reaction (PCR) was positive for *B. pertussis*. The patient was admitted to the Pediatric Intensive Care Unit (PICU) on 30th Apr, 2025 with a working diagnosis of malignant pertussis, based on severe lymphocytosis and respiratory compromise.

Respiratory support was initiated using high-flow nasal cannula (HFNC), and intravenous azithromycin (10 mg/kg/day) was started. Due to the elevated WBC count suggestive of pertussis-induced leukemoid reaction, hydroxyurea therapy was initiated at 20 mg/kg/day to prevent further leukostasis-related complications.

A pediatric hematology consultation was obtained, and other leukoreduction strategies, including leukapheresis and exchange transfusion, were discussed. However, given the patient’s hemodynamic stability, absence of pulmonary hypertension, and WBC count below the threshold typically requiring urgent leukoreduction (>50-100 × 10^9^/L), a shared decision was made to proceed with hydroxyurea as a non-invasive initial approach.

An echocardiogram was performed soon after PICU admission to assess for pulmonary hypertension and was reported as normal. Over the following five days, the infant showed marked clinical improvement with reduced work of breathing and declining oxygen requirements. HFNC was weaned over four days, followed by one day of nasal cannula oxygen support.

**Figure 2:**
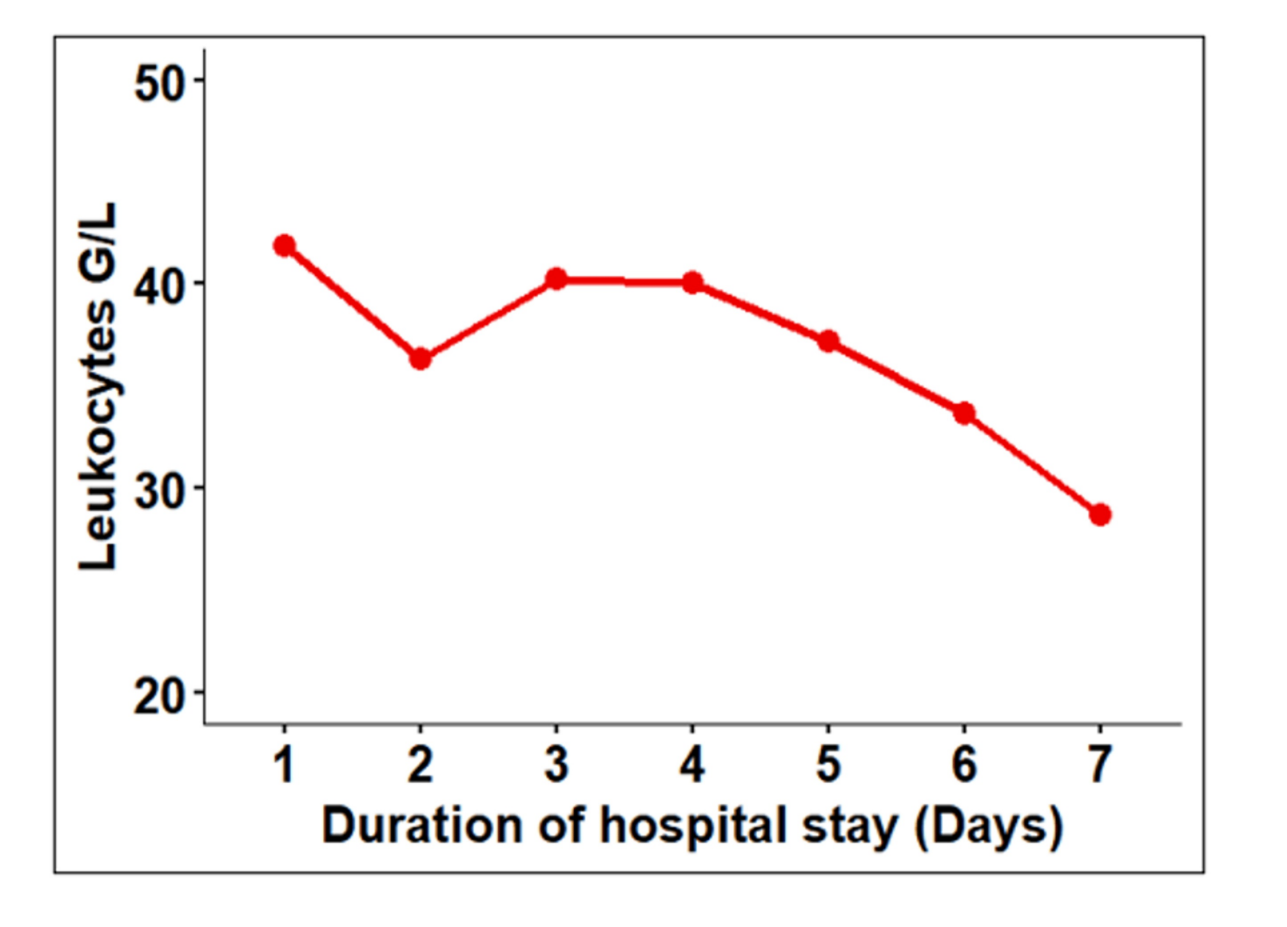
Change in total leukocytic count during hospital stay G/L: giga per liter (10^9^ cells/L)

The leukocyte count progressively decreased in response to hydroxyurea (Table 1 and Figure 2), and the patient remained hemodynamically stable.

**Table 1:** Trends in differential blood count and platelet count during the hospital stay G/L: giga per liter (10^9^ cells/L); Ref: reference range [8,9].

Day	Leukocytes G/L (Ref: 5–15)	Lymphocytes G/L (%) (Ref: 2–11 (40–80%))	Neutrophils G/L (%) (Ref: 1.5–8 (30–60%))	Monocytes G/L (%) (Ref: 0.2–1.0 (2–10%))	Eosinophils G/L (%) (Ref: 0–0.5 (0–5%))	Basophils G/L (%) (Ref: 0–0.2 (0–2%))	Platelets G/L (Ref: 150–450)
Day 1	41.82	32.25 (77)	8.00 (19)	1.38 (3)	0.05 (0.1)	0.14 (0.3)	791
Day 2	36.33	27.50 (76)	7.75 (21)	0.98 (2)	0.01 (0.1)	0.09 (0.2)	651
Day 3	40.21	31.57 (78)	6.70 (16)	1.84 (5)	0.06 (0.2)	0.04 (0.1)	710
Day 4	40.05	30.99 (77)	7.31 (18)	1.64 (4)	0.07 (0.3)	0.01 (0.04)	734
Day 5	37.20	28.97 (76)	6.30 (19)	1.66 (4)	0.16 (0.5)	0.11 (0.3)	707
Day 6	33.65	26.70 (77)	5.19 (15)	1.31 (4)	0.37 (0.7)	0.08 (0.2)	737
Day 7	28.70	21.50 (74)	5.50 (19)	1.40 (5)	0.30 (1)	0.10 (0.2)	683

No side effects of hydroxyurea were observed. The patient was monitored for hematologic suppression, gastrointestinal disturbances, and skin changes, all of which were absent.

The infant was transferred to the pediatric ward on 4th May, 2025 after completing a five-day course of azithromycin. Hydroxyurea was continued until day seven, at which point the WBC count had normalized. She was discharged in good condition after a total hospital stay of seven days.

## Discussion

Pathophysiology and risk factors

Malignant pertussis is partly attributed to the effects of pertussis toxin on the respiratory epithelium, leading to hypersecretions that obstruct small airways and increase pulmonary vascular resistance [6,10]. In addition, the pertussis toxin induces hyperleukocytosis, which can result in pulmonary vasculature sequestration, thrombosis, and subsequent organ failure [10,11]. The toxin also enhances platelet aggregation and vasoconstriction [12,13]. Moreover, it inactivates the Gi protein, resulting in cyclic adenosine monophosphate dysregulation, increased production of pro-inflammatory cytokines, and suppression of adaptive immune responses [12,13].

These mechanisms collectively contribute to a vicious cycle of ventilation-perfusion mismatch and pulmonary hypertension, with death typically resulting from cardiopulmonary failure [4,6,11,14]. Inactivation of the Gi proteins may also contribute to the increased heart and respiratory rates commonly observed in such cases [15], as was seen in our patient and previous case reports [4,16].

Although global vaccination efforts have reduced pertussis incidence, malignant pertussis continues to pose a serious threat to infants. Early identification of high-risk features is crucial due to the rapid and fatal course of the condition. Known risk factors for poor prognosis include age less than two months, low birth weight, prematurity, seizures during illness, leukocytosis >50 × 10^9^/L, and pulmonary hypertension [4,12,17]. Pulmonary hypertension, defined as a mean pulmonary artery pressure >20 mmHg on echocardiography, is especially associated with increased mortality. In our case, while pulmonary hypertension was absent, the leukocyte count was approaching critical thresholds, and timely intervention likely prevented further deterioration.

Current treatment modalities

First-line therapy for pertussis involves antibiotic treatment, usually with macrolides, to reduce disease severity and transmission [18]. However, rising resistance to macrolides has been reported [3]. Supportive respiratory care is essential, often involving high-flow oxygen or mechanical ventilation in severe cases.

In malignant pertussis, leukocytosis is a hallmark feature [15], with counts exceeding 50 × 10^9^/L particularly associated with pulmonary hypertension and increased mortality [4,11,19]. Leukocytosis leads to increased blood viscosity, which may result in thromboembolic complications, intracranial hemorrhage, and pulmonary hypertension [20].

When leukocyte levels are significantly elevated or accompanied by hemodynamic instability, leukoreductive interventions are often warranted. Available modalities include whole blood exchange and leukapheresis [21,22], both of which have demonstrated efficacy in improving oxygenation and reducing white cell burden. However, these procedures require specialized resources, central vascular access, and carry risks such as coagulopathy, electrolyte imbalances, and hemodynamic compromise [7,23,24].

Pulmonary hypertension, if present, may be managed with inhaled nitric oxide or phosphodiesterase inhibitors like sildenafil, although robust clinical data are lacking in this specific context.

Hydroxyurea as a therapeutic option

In our case, blood transfusion or leukapheresis was not pursued due to the patient’s relatively stable condition and absence of pulmonary hypertension. We opted for hydroxyurea, an oral chemotherapeutic agent approved for conditions such as chronic myeloproliferative disorders and sickle cell disease. Hydroxyurea works by inhibiting DNA synthesis, thereby reducing cell proliferation [25]. It also has anti-inflammatory effects and can modulate cytokine levels [26]. Furthermore, it promotes fetal hemoglobin production and induces nitric oxide release from endothelial cells [27].

There is limited but growing evidence supporting hydroxyurea's off-label use in malignant pertussis. Maitre et al. [6] described a 73-day-old preterm infant with malignant pertussis complicated by respiratory syncytial virus (RSV) infection. The patient was treated with hydroxyurea for five days instead of undergoing leukapheresis, and showed clinical improvement and leukocyte reduction.

Aldairi et al. [14] reported a case series of five infants diagnosed with malignant pertussis. In four of the cases, leukoreduction via exchange transfusion was used, while hydroxyurea was administered in one case. The patient treated with hydroxyurea showed full recovery, and the authors highlighted the importance of early recognition of leukocytosis and clinical deterioration using WBC and lymphocyte counts.

Blanc et al. [7] conducted a prospective case series of 27 infants with severe pertussis, 12 of whom exhibited malignant features. Hydroxyurea was used at 20 mg/kg/day, leading to a gradual leukocyte count reduction within seven days. The overall mortality rate among malignant pertussis cases was 25%; however, the study did not specify mortality rates among the subgroup treated with hydroxyurea. The authors also noted that hydroxyurea had a favorable safety profile compared to exchange transfusion, which may lead to thrombocytopenia. Hydroxyurea, however, did not impact platelet count [6,7].

In our patient, hydroxyurea was initiated early and continued for seven days. The leukocyte count showed a progressive decline during this period. The treatment course was shorter than in previously reported cases, likely due to early presentation and preemptive initiation of therapy. The patient was monitored for hydroxyurea-related toxicities, including complete blood count (for anemia, neutropenia, thrombocytopenia), liver and renal function, and gastrointestinal symptoms. No adverse effects were observed.

Summary and research gaps

This case supports hydroxyurea as a potential non-invasive therapeutic alternative for infants with malignant pertussis who are hemodynamically stable. It may allow gradual cytoreduction and avoid the risks associated with invasive procedures. However, current evidence is limited to case reports and small series.

There is an urgent need for randomized controlled trials to better define the safety profile, optimal timing, and dosing strategies for hydroxyurea in malignant pertussis [28]. While short-term use appears safe and effective, long-term adverse effects remain unknown. Until formal clinical guidelines are developed, hydroxyurea should be considered on a case-by-case basis, especially where conventional leukoreduction is unavailable or contraindicated.

## Conclusions

Hydroxyurea represents a promising treatment option in hemodynamically stable infants with malignant pertussis, capable of inducing a gradual yet effective decline in leukocytosis. While traditional leukoreductive modalities such as exchange transfusion and leukapheresis remain the mainstay in critically-ill infants, they are invasive, resource-intensive, and associated with procedural risks. Their implementation requires specialized expertise and equipment not readily available in many healthcare settings. In contrast, hydroxyurea offers a less invasive alternative that may be especially practical in low- and middle-income countries where access to leukapheresis is limited.

In the present case, hydroxyurea demonstrated a clinical benefit and favorable short-term safety profile. However, this observation is based on a single case report involving an infant with a leukocyte count below 50 × 10^9^/L and without pulmonary hypertension. Hydroxyurea was initiated preemptively based on the rising WBC trend, and its utility in more severe or decompensated cases remains uncertain, particularly due to its delayed onset of action.

The findings must be interpreted with caution, given the off-label use and limited generalizability of a single-patient experience. Larger prospective studies and multicenter data collection are needed to further evaluate hydroxyurea’s role, establish standardized dosing regimens, and assess its long-term safety in this population.
